# A multiplex PCR predictor for aCGH success of FFPE samples

**DOI:** 10.1038/sj.bjc.6602889

**Published:** 2005-12-06

**Authors:** E H van Beers, S A Joosse, M J Ligtenberg, R Fles, F B L Hogervorst, S Verhoef, P M Nederlof

**Affiliations:** 1 Division of Experimental Therapy, Netherlands Cancer Institute NKI/AvL, Plesmanlaan 121, 1066 CX Amsterdam, The Netherlands; 2 Department of Pathology and Human Genetics, Radboud University Nijmegen Medical Centre, PO Box 9101, 6500 HB Nijmegen, The Netherlands; 3 Division of Molecular Pathology, Netherlands Cancer Institute NKI/AvL, Plesmanlaan 121, 1066 CX Amsterdam, The Netherlands; 4 Family Cancer Clinic, Netherlands Cancer Institute NKI/AvL, Plesmanlaan 121 1066 CX Amsterdam, The Netherlands

**Keywords:** CGH, QC, FFPE archive, breast tumour

## Abstract

Formalin-fixed, paraffin-embedded (FFPE) tissue archives are the largest and longest time-spanning collections of patient material in pathology archives. Methods to disclose information with molecular techniques, such as array comparative genomic hybridisation (aCGH) have rapidly developed but are still not optimal. Array comparative genomic hybridisation is one efficient method for finding tumour suppressors and oncogenes in solid tumours, and also for classification of tumours. The fastest way of analysing large numbers of tumours is through the use of archival tissue samples with first, the huge advantage of larger median follow-up time of patients studied and second, the advantage of being able to locate and analyse multiple tumours, even across generations, from related individuals (families). Unfortunately, DNA from archival tissues is not always suitable for molecular analysis due to insufficient quality. Until now, this quality remained undefined. We report the optimisation of a genomic-DNA isolation procedure from FFPE pathology archives in combination with a subsequent multiplex PCR-based quality-control that simply identified all samples refractory to further DNA-based analyses.

Cancer cytogenetics has benefited greatly from the introduction of comparative genomic hybridisation (CGH) for mapping chromosomal gains and losses at a genome-wide scale (Kallioniemi *et al*, 1993; Gray *et al*, 1994). Subsequent development of the technique into array-CGH (also named matrix-CGH) has allowed increased automation, improved reproducibility and precision due to more accurate mapping of aberrations. This technology has been applied successfully to characterise congenital abnormalities at unprecedented precision (Veltman *et al*, 2002) and to characterise and classify tumours (Wessels *et al*, 2002; Nessling *et al*, 2005).

In most pathology laboratories, large archives of formalin-fixed, paraffin embedded (FFPE) material are often the only source of material for cancer research. It is our experience (Wessels *et al*, 2002; Van Beers *et al*, 2005) that a proportion of archival specimens appears unsuited for aCGH analysis, which is troublesome because array comparative genomic hybridisation (aCGH) experiments are tedious and expensive. In the past, we have noticed that this was not solved by repeating aCGH experiments, even when DNA was isolated from new sections from the same tissue blocks (Van Beers *et al*, 2005). Nevertheless, it is possible to obtain high-quality data using archival DNA samples in array CGH experiments (Figure 1) (Gray *et al*, 1994; Ried *et al*, 1995; Albertson and Pinkel, 2003; Heidenblad *et al*, 2004; Loo *et al*, 2004; Devries *et al*, 2005), even from 20-year-old tissue blocks, provided that robust procedures, high-quality reagents and ‘good’ sample DNA quality are being used. A ‘good sample quality’ definition and an assay to determine this FFPE DNA sample quality would therefore be of great value.

Molecular biological assays, including aCGH on FFPE archival specimens, would be more efficient when good and bad quality samples were identified prior to aCGH assays, by a quick, cheap, simple and reliable assay. Variability, mostly in sample fixation (time), and also duration of storage affect DNA quality. Improvements in many pathology laboratories in sample handling, including shortening of the fixation duration to 24–48 h and using buffered formalin may have contributed to the increased quality of tissue-extracted DNA (Legrand *et al*, 2002). In an attempt to predict the success of aCGH hybridisation, many laboratories have assessed DNA quality by DNA gel electrophoresis. Although such analyses provide information on the size, amount and distribution of the fragment sizes of the (partially) degraded DNA, this did not correlate well with aCGH success in our hands. Our hypothesis is that apart from the fragment length, DNA crosslinks caused by fixation are of major importance for hybridisation results. We therefore focused on improvement of the DNA isolation method to reduce DNA crosslinks, and on an assay to determine the abundance of DNA crosslinks as a measure of DNA quality. This prompted us to evaluate retrospectively our good and bad aCGH experiments and devise a method that indicates DNA quality and aCGH success. This resulted in a modified DNA isolation method and a quality test using a multiplex-PCR assay for sample DNA quality control together with measurement of specific labeling of Cyanin *cis*-platinum-labelled nucleotides in the test DNA.

## MATERIALS AND METHODS

### DNA isolation

Genomic DNA was isolated from 10 10 *μ*m-thick paraffin-embedded tissue sections. Sections were deparaffinated twice for 5 min in xylene, rehydrated in 100, 96 and 70% ethanol for 30 s each, stained with haematoxylin for 30 s, rinsed with water and incubated overnight in 1 M NaSCN at 37°C to remove crosslinks. Slides were rinsed twice 10 min in 1 × PBS at room temperature, and completely air-dried. Tumour tissue was scraped from the glass with a scalpel to obtain at least 70% tumour cells in 200 *μ*l Qiagen ATL buffer (QIAamp® DNA extraction kit cat. 51306), transferred to eppendorf tubes and incubated with 27 *μ*l proteinase-K (20 mg/ml stock) at 450 rpm (Eppendorf® Thermomixer R) at 55°C. Three more aliquots of 27 *μ*l proteinase-K were added at 4, 20 and 28 h. After a total protK incubation of ∼44 h, DNA isolation proceeded as in the manufacturer's protocol (Qiagen, Cat. 51306). Samples of isolated genomic DNA were analysed by 0.8% agarose gel electrophoresis to visualise DNA concentration and size distribution. In case of tumour tissue, we scraped regions containing at least 70% tumour as indicated by an experienced breast cancer pathologist. aCGH reference DNA was isolated from peripheral blood lymphocytes from six apparently healthy female individuals. It was pooled and sonicated until its median fragment length was similar to that of the test samples.

### Multiplex PCR

We analysed 100 ng as measured by optical density at 260/280 nm of each archival genomic DNA sample by a multiplex PCR. The PCR reaction was performed with four primer sets that produce 100, 200, 300 and 400 bp fragments from nonoverlapping target sites in the GAPDH gene (chr12) in 30 *μ*l with final concentrations: 0.133 *μ*M of each of the following eight 5′–3′primers: 100F gttccaatatgattccaccc; 100R ctcctggaagatggtgatgg; 200F aggtggagcgaggctagc; 200R ttttgcggtggaaatgtcct; 300F aggtgagacattcttgctgg; 300R tccactaaccagtcagcgtc; 400F acagtccatgccatcactgc and 400R gcttgacaaagtggtcgttg in a reaction with 10 mM Tris-HCl pH 8.8, 1.5 mM MgCl_2_, 75 mM KCl, 0.2 mM dNTPs, 1 U *Taq* DNA-polymerase (Invitrogen cat. 18038-26). PCR was performed in thin-wall tubes in an MJ Research PCR apparatus for 4 min 94°C, 35 cycles each of 1 min 94°C, 1 min 56°C and 3 min 72°C, followed by 7 min 72°C ending at 15°C. After addition of 6 *μ*l (5 ×) loading dye, 10 *μ*l of each sample was analysed on a 1.5% TBE agarose ethidium bromide-stained gel. Samples were classified based on the largest of four possible PCR products detected, namely 100, 200, 300 and 400 bp. The GAPDH genomic target for amplification is more or less arbitrary but the lengths of the products were purposely chosen based on earlier experience with FFPE DNA amplification (MJL, unpublished results).

### Genomic DNA labelling

All labelling reactions were performed with the Cy3 and Cy5 conjugates from the Universal Linkage System (ULS, Kreatech Biotechnology, Amsterdam the Netherlands) (Raap *et al*, 2004) Labelling efficiency for ULS-Cy3 and ULS-Cy5 were calculated from *A*_260_ (DNA), *A*_280_ (protein), *A*_550_ (Cy3) and *A*_649_ (Cy5) after removal of unbound ULS, on a NanoDrop®ND-1000 spectrophotometer (NanoDrop Technologies, Wilmington, DE, USA). The degree of labeling (DOL) was calculated from the specific molar extinction ratios for Cy3, Cy5 and DNA and must be between 1 and 4% (between 1 and 4 ULS molecules per 100 bp) for optimal hybridisation signals.

### Array CGH

The human 3600 BAC/PAC genomic clone set, covering the full genome at 1 Mb spacing used for the production of our arrays, was obtained from the Welcome Trust Sanger Institute (http://www.sanger.ac.uk/). Information on this clone set can be obtained at the BAC/PAC Resources Center Web Site (http://bacpac.chori.org). Degenerate oligonucleotide PCR-products from all BAC clones were prepared for spotting on CodeLink™ Activated Slides (Amersham Biosciences, Prod. No. 300011 00) according to detailed protocols (Pinkel *et al*, 1998) with some modifications (Alers *et al*, 1999). All clones (three replicates for each probe) were spotted in randomised fashion across 48 subarrays, each containing 270 spots and hybridised for 48–72 h at 37°C on an orbital shaker (300 rpm) in a humidified chamber with 2 *μ*g tumour-DNA labelled with ULS-Cy5 and 2 *μ*g sonicated lymphocyte control DNA labelled with ULS-Cy3. After washing, arrays were scanned on a Microarray Scanner (G2505B Agilent Technologies), and spots quantified with ImaGene® software (version 6.0.1 BioDiscovery, Marina Del Rey, CA, USA). Computation of the profiles included local background subtraction, Cy5/Cy3 ratio, ^2^log-transformation and subarray normalisation to its median. The ^2^log ratios for all nonflagged spots are then plotted (Figure 2D) along with the standard deviation for each triplicate as smaller dots (red) closer to the *X*-axis using the secondary *y*-scale to the right. Bad morphology or uniformity spots were flagged in ImaGene®. When flagged spots accounted for >5% of all spots, hybridisations were excluded. The BAC clones are ordered by position as assigned by NCBI-Build35 (http://genome.ucsc.edu/cgi-bin/hgGateway) in the genome beginning at the telomere of 1p and ending at the telomere of Yq.

## RESULTS AND DISCUSSION

In a systematic approach, we have identified and optimised the selection steps for FFPE archival material to be used in downstream applications, particularly for aCGH.

### Formalin-fixed, paraffin-embedded archival tissue DNA quality

**Figure 1:**
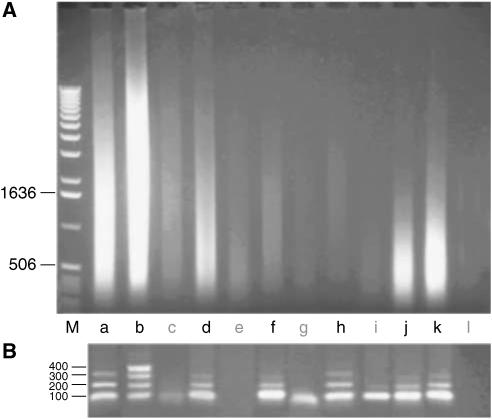
A total of 12 unselected DNA preparations from FFPE breast tumours with corresponding multiplex PCR quality controls. (**A**) DNA was isolated from archival blocks stored between 6 (lane b) and 29 (lane l) years. Lane M indicates the molecular size standard (bp). Sample a through l were fixed and stored 11, 6, 22, 20, 18, 11, 8, 7, 19, 17, 16 and 29 years ago, respectively. Lanes in bold a, b, d, f, h, j and k indicate samples with successful aCGH. The oldest samples in this panel successful in aCGH are in lanes d, k and j (20, 17 and 16 years). (**B**) Agarose gel showing multiplex PCR product sizes in bp (see Materials and Methods) for the corresponding samples above.

In the past, we have used size and size distribution of genomic DNA as a surrogate quality end point. The resulting aCGH profiles were sometimes inconsistent with the estimated sample quality. Figure 1A shows a typical series of 12 isolated genomic DNA samples from FFPE tissue sections. Each lane contains 5 *μ*l (10%) of each isolate. The oldest sample was embedded and stored 26 years before DNA extraction (lane L). The amount of DNA is variable due to the variability in number of nuclei, and dependent on size of the tissue scraped. Furthermore, Figure 1A shows that genomic DNA from archival tissue is severely fragmented with an estimated median DNA fragment size often below 1 kb and varies substantially between samples (cf. lanes B *vs* J). In addition, we observed variability in the size distribution (i.e. long *vs* short smear) between samples (cf. lanes B *vs* J).

### Multiplex PCR quality assay

**Figure 2:**
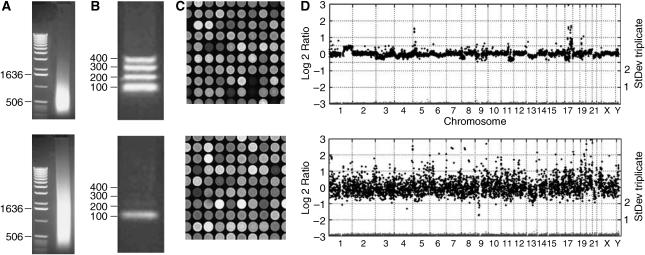
aCGH success is determined by the ability to PCR-amplify fragments of > 100 bp from the sample (FFPE) DNA template. (**A**) 0.8% agarose gel electrophoresis shows amount, size and smear-lengths of sample DNA isolated from FFPE tumour tissues. (**B**) Multiplex-PCR reveals whether a 100, 200, 300 or 400 bp fragment are amplified from 100 ng total genomic DNA. (**C**) Representative partial images of array CGH hybridisations. Array CGH was performed on 3500, DOP-amplified BAC-DNA microarrays (see Materials and Methods) printed on Codelink® slides. (**D**) Gain and loss profiles were plotted where the ordinate represents the log 2 ratio for the mean of triplicates for each BAC, and abscissa the mapping on the genome (from chromosome 1 to Y, left to right). In red, the standard deviation of the triplicate measurements is plotted to a secondary *Y*-axis on the right.

An unknown fraction of these samples are refractory to molecular assays including aCGH. The challenge was to identify these samples before performing aCGH. We hypothesised that FFPE samples even after de-crosslinking may still contain DNA crosslinks that prevent specific hybridisation and therefore render the sample useless for aCGH. We assumed that with increasing occurrence of DNA crosslinks, the 400, 300, 200 and 100 bp PCR products would become less abundant or even disappear in that order. We thus used the relative amounts of the four possible PCR products as a reporter of DNA quality, and therefore suitability in aCGH. Our quality assay requires 100 ng genomic DNA of each sample in a single multiplex PCR reaction. Representative archival DNA preparations are shown in Figure 1. Two samples (e and l) failed to produce the 100 bp PCR fragment (Figure 1B) and were not successful in subsequent aCGH. Three samples (c, g, i) only produced the 100 bp fragment and each failed in aCGH. All seven samples with a PCR displaying fragments of 200 bp or more were successful in aCGH. Then, we tested DNA samples retrospectively for cases (*N*=26) (Table 1) with known aCGH outcome. We found a good correlation between the ability to obtain PCR products and the quality of the aCGH experiment. There were 24 samples with PCR product and two without (Table 1). The two samples without PCR product as well as two out of three samples with the 100 bp PCR fragment only were not successful in aCGH (cf. Figure 2D lower panel). All samples with a 200 bp or greater size PCR fragments resulted in successful aCGH profiles (cf. Figure 2D upper panel). Then, in a prognostic approach, we used the multiplex assay outcome to decide when to perform aCGH, that is, aCGH was only performed if a sample had at least the 100 bp PCR fragment (83 of 93 samples). Only six of 37 (16%) samples that had the 100 bp as largest PCR product resulted in good aCGH results. For the samples with 200 bp as the largest product, 38 of 39 (97%) resulted in good aCGH profiles. All seven samples with a 300 or 400 bp products were successful in subsequent aCGH. These results indicate that samples without a 100 bp fragment should not be used in aCGH and that DNA samples with amplification of the 200 bp fragment or larger seem to be of sufficient quality for aCGH analysis.

### aCGH profiles for FFPE breast tumour samples

Figure 2 illustrates our findings on aspects of DNA quality *vs* aCGH success. All four upper panels represent a good quality archival DNA sample, whereas the four lower panels represent a poor quality archival DNA sample. Both panels A show the amount and fragment size distribution for these samples after isolation without further restriction digestion. Even though the DNA fragments from the lower sample are somewhat smaller, both DNA samples theoretically consist of appropriately sized fragments for aCGH. Both panels B show the result of multiplex quality control PCR using 100 ng of input DNA. It is here, and only here, that we detect the crucial difference between good and bad samples, defined as a minimum of 200 bp amplifiable target sequence. Both panels C show the resulting hybridisation and are highly similar in quality (area shown is not the same for both arrays). Finally, panels D show gains (positive log ratios) and losses (negative log ratios) of (parts of) chromosomal material in the breast tumours. The upper panel shows a successful aCGH experiment whereas the lower panel represents a ‘noisy’ and therefore useless aCGH. Each black dot of the profile represents the mean of three replicates on the same array (triplicate) and the standard deviation of the replicate is plotted below to a secondary *Y*-scale on the right. Most standard deviations are well below 0.2 and many below 0.1, which indicate very reproducible hybridisations for the good but notably also for the bad DNA sample. The decisive difference between good and bad samples that can be easily scored is the presence of the 200 bp multiplex PCR fragment.

### DNA quality from three pathology archives across three decades

With our DNA isolation protocol, we were able to obtain high-quality DNA from the majority of samples from different pathology archival paraffin blocks as old as 25 years. An independent estimation of DNA quality in FFPE samples that almost entirely consisted of samples from our own institute was calculated using a different PCR, in this case generating a 157 bp fragment on 1345 samples, 1264 (94%) of which were positive in this PCR. We found no evidence for different success rates of the 157 bp PRC using samples fixed during the last 25 years studied, whereas DNA from samples fixed before 1970 was often problematic defined by the failure to produce the 157 bp PCR fragment (results not shown). There were 202 of 246 (82%) positive PCR reactions in samples fixed between 1970 and 1980, 666 of 682 (97%) samples fixed between 1980 and 1990, and 397 of 418 (95%) fixed after 1990 (M Schmidt, NKI/AvL personal communication).

There appeared to be a surprisingly large difference between the archives that we sampled. We then compared the multiplex PCR quality assessment across three FFPE breast cancer sample series mentioned in this study, that is, 26 retrospective samples, 93 prospective samples and, the independent study of 1345 breast cancer samples for which PCR success rates were 85, 55 and 94%, respectively. Although, the latter percentage (94%) in this comparison is undoubtedly an overestimation due to the fact that it is only analysed for production of a 157 bp fragment compared to 200 bp fragment in the other two series, it seems that no *a priori* success rate can be assumed when different archives are being sampled.

### *Dpn*II digestion or not?

Array CGH requires that high molecular weight genomic DNA is fragmented to an appropriate fragment size (e.g. by sonication or restriction digestion). Fragmentation can be omitted for aCGH when DNA is isolated from FFPE archival material since it is already fragmented. We compared array CGH using archival DNA with and without prior *Dpn*II restriction and found similar results (data not shown). As expected, DNA gel electrophoresis of archival DNA samples clearly showed the typically fragmented DNA for FFPE samples (Figure 1) explaining why restriction digestion is unnecessary on such samples.

## CONCLUSION

Since concentration and size distribution (as assessed by ethidium bromide agarose gel electrophoresis) of genomic DNA isolated from FFPE tissue are inadequate predictors *per se* for aCGH success, we have developed a method for DNA isolation from FFPE tissue with a subsequent simple and reliable multiplex PCR protocol that predicted successful aCGH with high accuracy. Of our archival samples, 11% (12 out of 107) proved unsuitable for any of the four PCR products and were refractory to aCGH analysis. Furthermore, when genomic DNA was reisolated from adjacent serial sections of those paraffin tissue blocks that failed the multiplex PCR test and aCGH, both multiplex PCR and aCGH results remained unchanged indicating that DNA suitability for aCGH seems intrinsic to the embedded tissue and is probably related to tissue treatment and duration of storage. Finally, the 157 bp product PCR was used to assess the quality of a much larger set of 1345 DNA samples isolated from three independent pathology archives from samples fixed between 1970 and present. This series was positive for the 157 bp PCR in 94% of the cases, suggesting that aCGH should be widely applicable to archival samples when isolated and selected as indicated above.

## Figures and Tables

**Table 1:** Correlation between PCR result and subsequent successful array comparative genomic hybridisation (aCGH)

**(A) Retrospective correlation of 26 breast tumour formalin-fixed, paraffin-embedded (FFPE) DNA samples aCGH success with performance of their multiplex PCR**					
**DNA quality *vs* aCGH**	**Success (%)**	**Good aCGH**	**Failed aCGH**	***N***	
400 bp	100	11		11	
300 bp	100	8		8	
200 bp	100	2		2	
100 bp	33	1	2	3	
No product	0	—	2	2	
					
Totals		22	4	26	
					
**(B) Prospective correlation of 93 breast tumour FFPE DNA samples aCGH success with performance of their prior multiplex PCR**					
**Largest product in multiplex PCR**	**Success (%)**	**Good aCGH**	**Failed aCGH**	**Not done**	***N***
400 bp (100%)	100	2	0		2
300 bp	100	5	0		5
200 bp	97	38	1		39
100 bp	16	6	31		37
No product	ND	0	0	10	10
					
Totals		51	32	10	93
